# A Review of Polymeric Micelles and Their Applications

**DOI:** 10.3390/polym14122510

**Published:** 2022-06-20

**Authors:** Suguna Perumal, Raji Atchudan, Wonmok Lee

**Affiliations:** 1 Department of Chemistry, Sejong University, Seoul 143747, Korea; wonmoklee@sejong.ac.kr; 2 School of Chemical Engineering, Yeungnam University, Gyeongsan 38541, Korea; 3 Department of Chemistry, Saveetha School of Engineering, Saveetha Institute of Medical and Technical Sciences, Chennai 602105, TN, India

**Keywords:** polymers, critical micelle concentration, micelles, drug delivery, imaging

## Abstract

Self-assembly of amphiphilic polymers with hydrophilic and hydrophobic units results in micelles (polymeric nanoparticles), where polymer concentrations are above critical micelle concentrations (CMCs). Recently, micelles with metal nanoparticles (MNPs) have been utilized in many bio-applications because of their excellent biocompatibility, pharmacokinetics, adhesion to biosurfaces, targetability, and longevity. The size of the micelles is in the range of 10 to 100 nm, and different shapes of micelles have been developed for applications. Micelles have been focused recently on bio-applications because of their unique properties, size, shape, and biocompatibility, which enhance drug loading and target release in a controlled manner. This review focused on how CMC has been calculated using various techniques. Further, micelle importance is explained briefly, different types and shapes of micelles are discussed, and further extensions for the application of micelles are addressed. In the summary and outlook, points that need focus in future research on micelles are discussed. This will help researchers in the development of micelles for different applications.

## 1. Introduction

### 1.1. Why and When Does Self-Assembly Occur?

Self-assembly is everywhere in nature, and we observe it in our daily lives. Self-assembly is a process in which an organization in structure takes place spontaneously. Self-organized structures are available in nature, such as those seen in honeycombs, patterns in butterfly wings, dunes in the desert, patterns/stripes on zebras, the human brain structure, spider webs, fingerprints, patterns of DNA, phospholipids in the cell wall structure, and so on. In science, small molecules aggregate or self-assemble into specific systems, either in bulk or in solution. This arrangement exhibits higher stability due to its physical and mechanical properties. Many reports are available regarding the self-assembly of surfactants and polymers. in particular, polymers, such as amphiphilic block copolymers, graft polymers, and cyclic polymers, show various architectures [1,2,3]. Apart from polymers, polyelectrolytes [4,5,6] and phospholipids [7,8,9] are used for the formation of micelles, but here, micelles with amphiphilic polymers are the focus.

In a diluted state, polymers will be present as dispersed polymer units in a medium. At a certain concentration, the polymers will tend to arrange themselves in an ordered structure, and this concentration is named “critical micelle concentration” (CMC) [10,11]. CMC can be examined by surface-tension measurements, and the surface tension dramatically changes with the concentration of the molecules in the medium [12]. Thus, the concentration at which the surface tension value does not change is marked as CMC [13,14,15]. The products of the self-assembly of amphiphilic polymers or the spontaneous aggregation of amphiphilic polymers are called “micelles” and “vesicles”. Micelles and vesicles play vital roles in various applications. Micelles are monolayers of self-assembled polymers, while vesicles are bilayers of self-assembled polymers. Many reports and reviews are available on vesicles [16,17,18,19,20,21,22]. Thus, only polymeric micelles are the focus here; for the formation of the micelle, CMC plays an important role. Thus, the determination of CMC is focused on; then, the formation of micelles is discussed in detail. Further, the applications of micelles are deliberately explained.

### 1.2. Determination of CMC

Generally, CMCs are measured using various methods, such as the surface-tension method [23,24], light-scattering method [25], electric conductivity method [26], osmotic pressure method [26,27], surface plasmon resonance method [23], and fluorometric method [24,25]. The light-scattering method can be used to determine the CMCs of polymers. Upon CMC formation, a sudden increase in the intensity of scattered light will be observed [25,28]. Dielectric constant measurements, consisting of the Philips method, permit the determination of CMC [29,30]. Chromophores, such as pyrene, are used for the determination of CMC by the fluorometric method [25]. However, fluorometric methods cannot be used to quantify the CMC of surfactants that have very low CMC values [24]. Among these methods, conductivity and surface-tension are suitable methods for the calculation of the CMCs of surfactants or polymers with high CMC values [24]. Here, a few efficient and simple methods for calculating CMCs are discussed in detail.

CMC calculations using surface tension were determined by a concentration series. The surface tension of surfactants or polymers with different concentrations were measured. The surface tension is linearly dependent on the logarithm’s polymer or surfactant concentrations. However, above the CMC, the surface tension is independent of the polymer’s or surfactant’s concentration. Typically, the CMC is the intersection point between two slopes. The intersection points are deteriorations of the straight line of the linearly dependent region and a straight line passing through the flat terrain where no change in surface tension has been observed. The surfactant CMC was determined using surface plasmon resonance of silver nanoparticles [23]. Trujillo et al. [12] discussed the determination of the CMC of cavitand additives (vase-shaped molecular receptors) by capillary method, Wilhelmy plate, filter method, and dye micellization [31,32,33,34,35,36].

Generally, the meniscus of the solution depends on the type of solution; some solutions will cause a depression in the meniscus (such as water), and some solutions will cause an elevation in the meniscus (such as mercury). In the capillary method, concerning the surface tension, the depression or elevation of the solution in the capillary tube will be observed; thereby, the surface tension is measured [33]. The Wilhelmy plate method is named after German chemist Ludwig Wilhelmy. This method enables the direct and indirect measurement of the adhesion tension and the contact angle, respectively. The plate, immersed in the solution, is retracted with applied force. Thus, the surface tension of the solution is measured [32,33]. For the filter method, instead of using a plate, filter papers are used as a part of the Wilhelmy method [35]. In dye micellization, specific dyes are used to determine the CMCs of surfactants or polymers. Shifts in the maximum wavelength of the solution of polymers or surfactants at a specific wavelength are observed [12,24,35].

**Figure 1:**
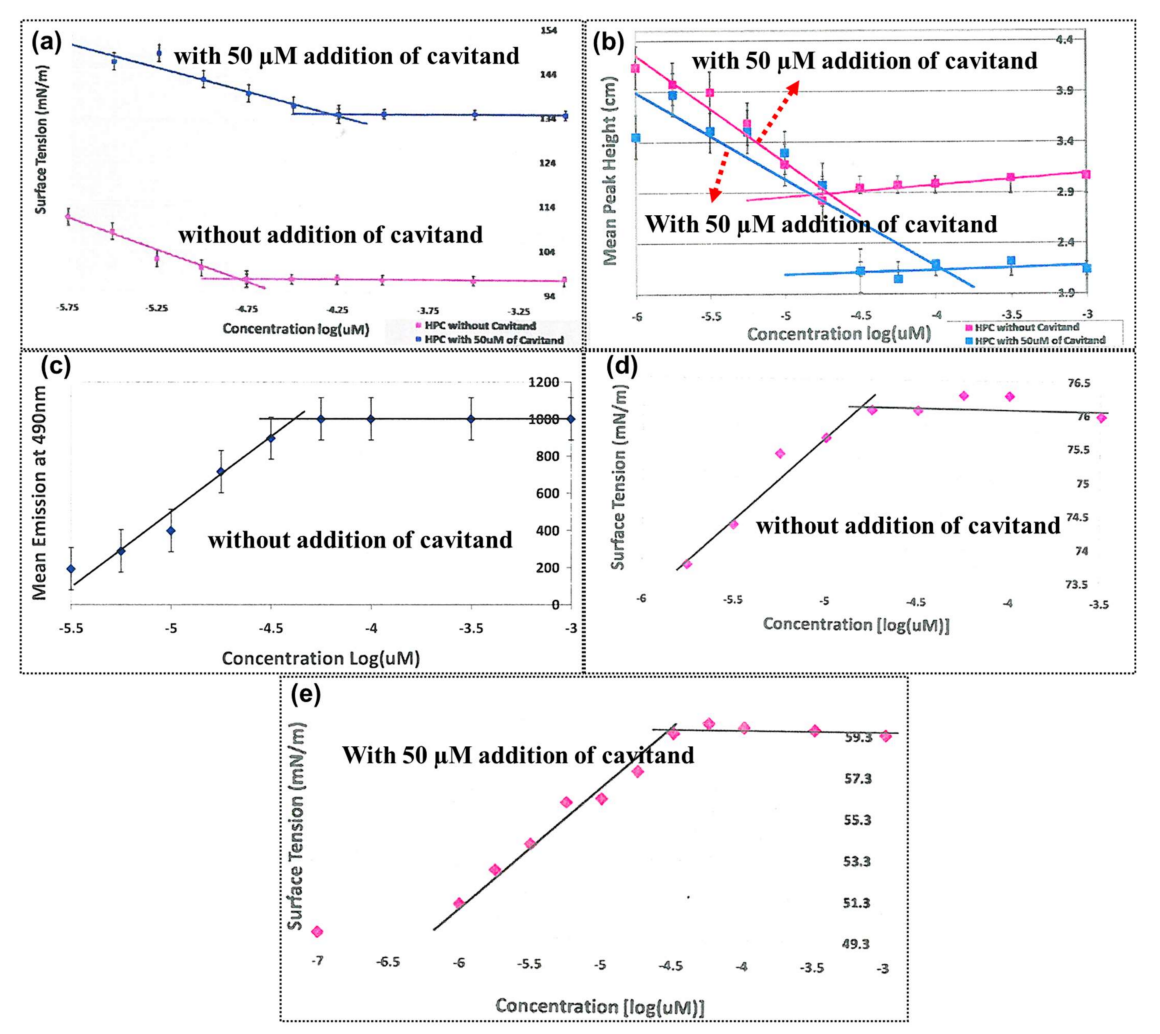
CMC determination of HePC with 50 µM of cavitand and without cavitand via the filter paper method (**a**), the capillary height method (**b**), the dye micellization method (**c**), and the Wilhelmy plate method (**d**,**e**). Reprinted from ref. [12].

Figure 1 shows the measurement of the CMC of hexadecylphosphocholine (HePC) with the addition of cavitand additives via different methods. Without a cavitand additive, the CMC of HePC was calculated as 17.8 µM, and it increased to 31.6 µM with the addition of 50 µM of cavitand via the filter paper (Figure 1a) method. Figure 1b shows the results obtained by the capillary height method; Figure 1c shows results obtained by the dye micellization method; and Figure 1d,e shows the results obtained by the Wilhelmy plate method. CMC by dye micellization was determined only without the addition of a cavitand additive; the CMC obtainable with an additive could not be determined. All methods reveal the same values for the CMC [12]. Thus, these are effective methods for the determination of CMCs. Thereafter, the researchers adopted a convenient method for the determination of CMC.

**Figure 2:**
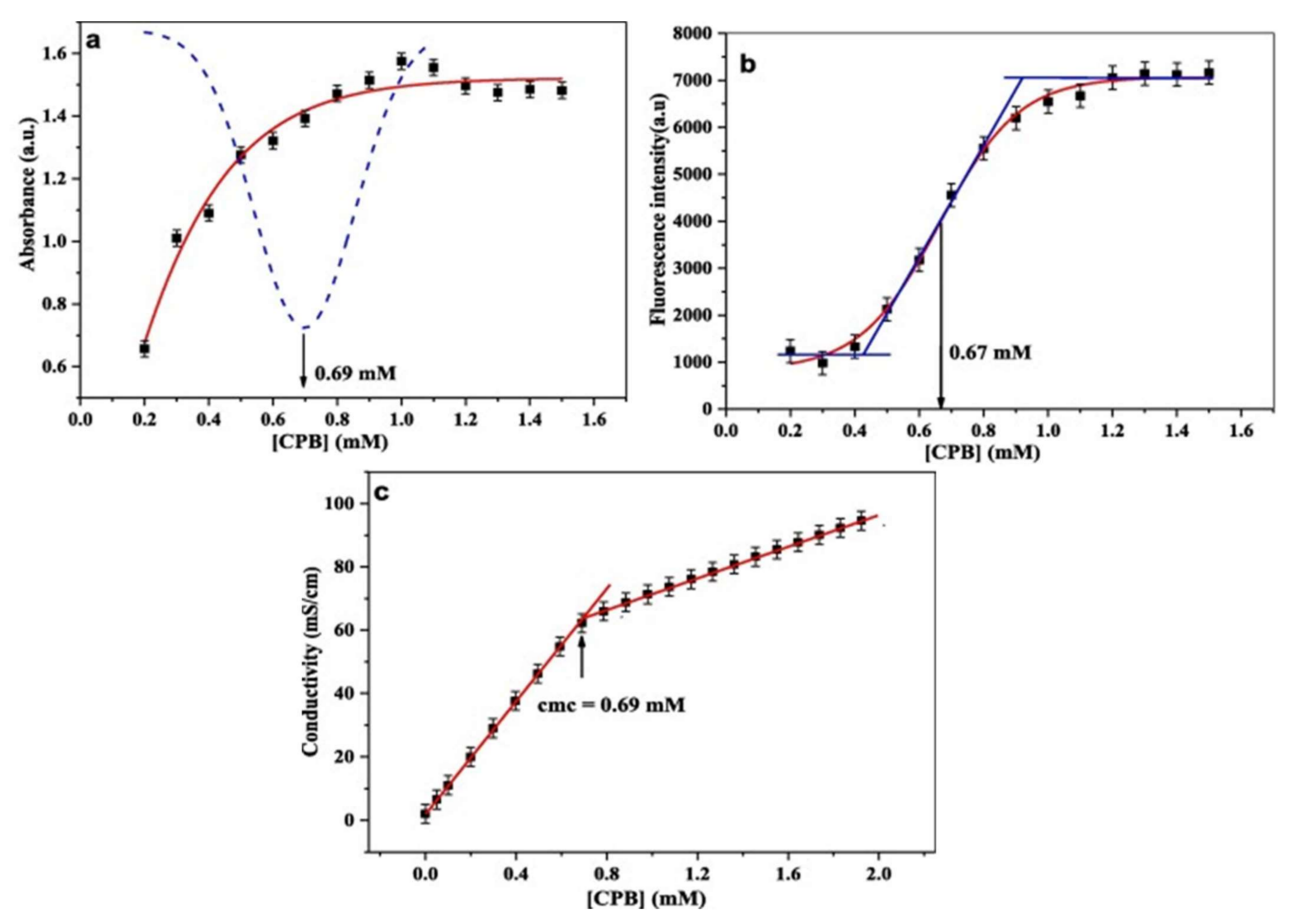
CMC measurement of CPB using absorbance (**a**), fluorescence (**b**), and conductivity (**c**) as related to concentration of CPB. Reprinted from [36], Copyright (2019) with permission from Elsevier.

Using a fluorescein probe with different surfactants, such as *N*-cetylpyridinium bromide monohydrate (CPB), sodium bis (2-ethylhexyl) sulfosuccinate, and *N, N*-dimethyltetradecylamine *N*-oxide, and using absorbance and fluorescence techniques, one can measure CMCs. The absorbance intensity is plotted with a concentration of CPB; the breakpoint observed in sigmoidal fit behavior is referred to as the CMC (Figure 2a). This CMC value was further confirmed by fluorescence (Figure 2b) and conductivity measurements (Figure 2c), where fluorescence intensity and conductivity with different concentrations were plotted, respectively. The value of the CMC obtained by absorbance was calculated as 0.69 mM and showed good agreement with the CMCs obtained via fluorescence (0.67 mM) and conductivity (0.69 nM) measurements [37].

## 2. Polymeric Micelles

Micelles are formed by the self-assembly of amphiphilic polymers at the CMC [36,37,38,39]. This self-assembly of an amphiphilic polymer with a hydrophobic tail and hydrophilic head is referred to as a polymeric micelle [40,41,42,43]. Depending on the hydrophobic and hydrophilic segments and solvent conditions, the morphologies of micelles take different shapes, including spheres, tubules, inverse micelles, bottle-brush shapes, and so on [44]. Micelles are prepared by various methods, such as dilution [44,45,46], lyophilization [46,47], solvent evaporation [48,49,50], dialysis [48,51,52,53], and oil-in-water emulsion. Block copolymers [53], random block copolymers, and grafted polymers are self-assembled into micelles. The micelles are characterized using various techniques, including atomic force microscopy (AFM) [53], small-angle X-ray scattering (SAXS), small-angle neutron scattering (SANS), transmission electron microscopy (TEM), dynamic light scattering (DLS), and electron paramagnetic resonance (EPR) spectroscopy.

### 2.1. Polymeric Spherical Micelles

**Scheme 1:**
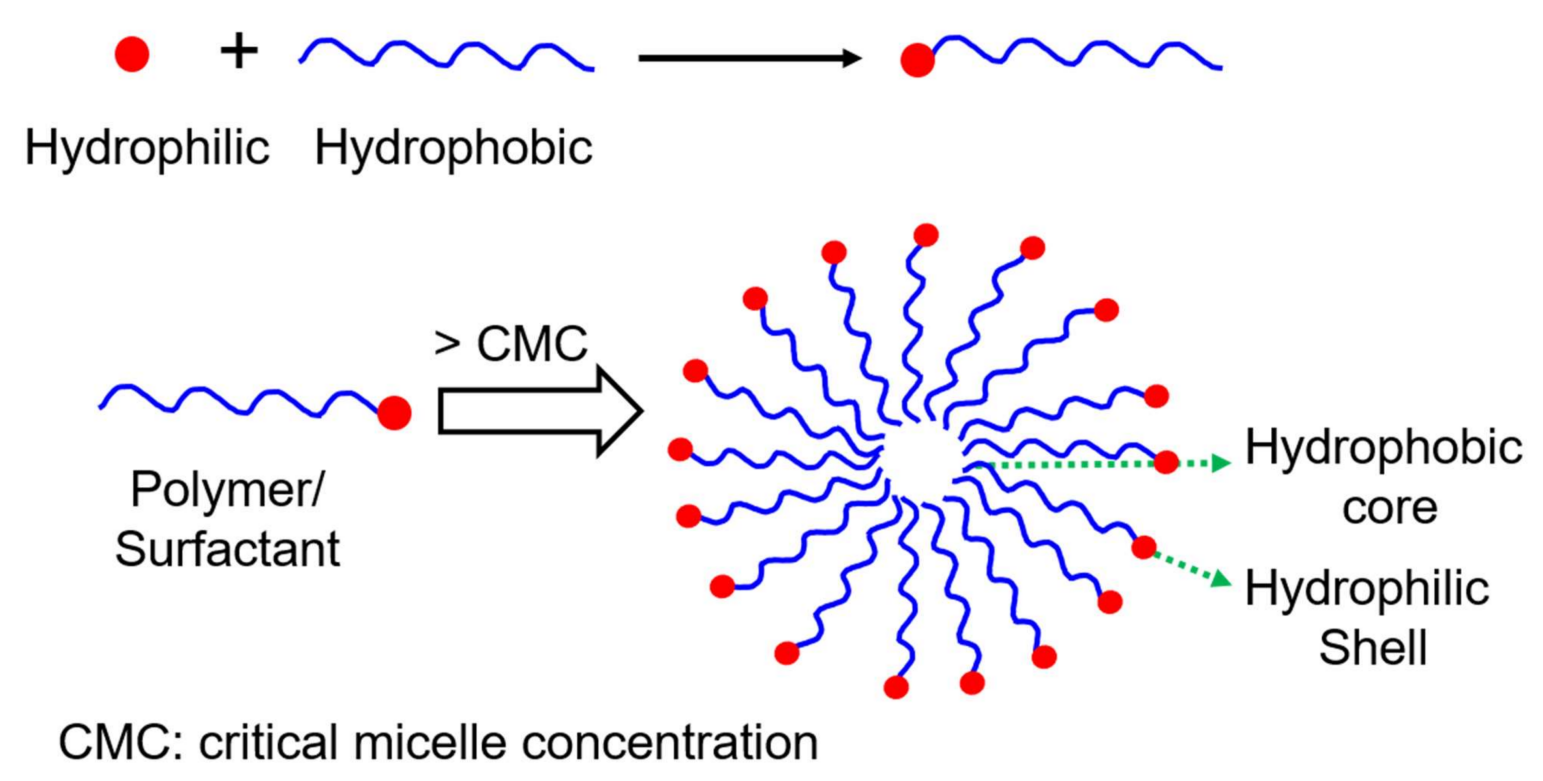
Illustration of the self-assembly of the polymer or surfactant into a spherical micelle.

Above the CMC, the amphiphilic polymers self-assemble into spherical micelles. The hydrophobic tail aggregates as the inner core and the brush-like structure of the hydrophilic head unite as a shell as described in Scheme 1 [54]. This hydrophobic core can accommodate hydrophobic drugs via hydrophobic interactions. The shell of micelles, which has hydrophilic units, will interact with water molecules surrounding the micelle. This helps in the stabilization of prepared micelles in an aqueous solution [55,56,57,58]. In this section, the preparation and characterization of polymeric micelles are discussed.

**Figure 3:**
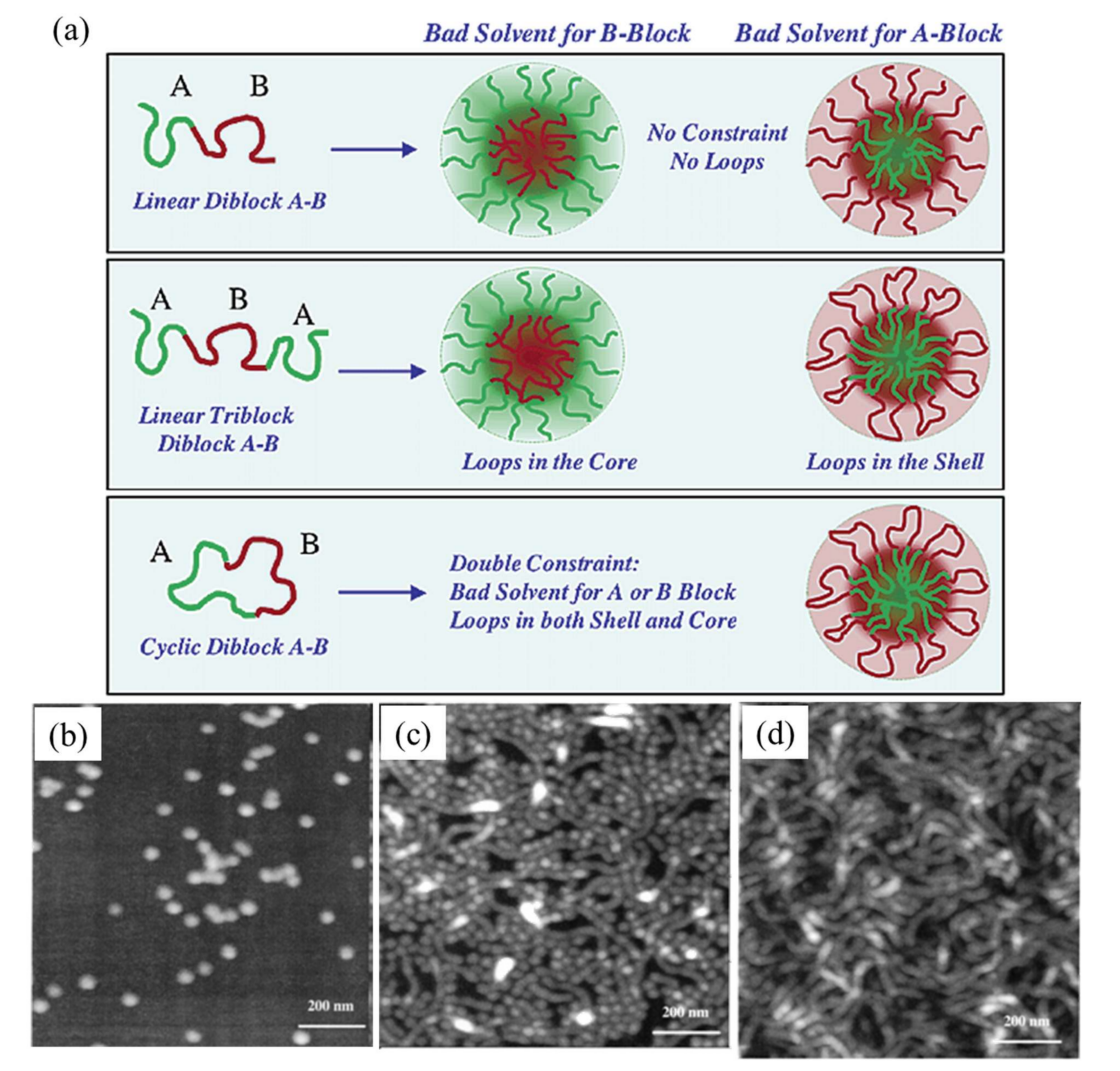
(**a**) schematic representation of topological constraints on the micelle using linear diblock (A-B), linear triblock (A-B-A), and cyclic diblock (A-B) polymers. AFM topographic images of PS-PI micelles in heptane at concentrations (**b**) 0.1 mg/mL, (**c**) 1 mg/mL, and (**d**) 5 mg/mL. Reprinted with permission from [53]. Copyright 2003 American Chemical Society.

Poly(*N*-vinylpyrrolidone)-block-poly(vinyl acetate) (PVP-b-PVAc) is a polymer that was prepared by reversible addition−fragmentation chain-transfer (RAFT) polymerization. PVP-b-PVAc micelles were prepared by the dialysis method [59]. Recently, preparation of micelles was reported using random block copolymers using poly(ethylene glycol) (PEG), methyl ether methacrylate, and butyl methacrylate (BMA). Polymer chain exchange between micelles in water was successful where micelles were prepared using the solvent evaporation method [50]. Tough micelles are prepared using diblock copolymers, polyethyleneoxide, and polyethylethylene. These micelles are tougher than phospholipid bilayers and show 10 times less permeable water than phospholipid bilayers [60]. Linear poly(styrene-b-isoprene) (PS-PI) was transformed into the cyclic structure by the effect of heptane for the PI block [53]. Further, the shapes and sizes of linear and cyclic polymers are affected by micelle concentrations. Figure 3a advice for the selection of solvent results in a different structure. Good solvents of B blocks in ABA tri-copolymer form micelles with loops in the core. Cyclic diblock AB results in the loop, core, and shell as shown in Figure 3. At low concentrations (0.1 mg/mL) of PS-PI, the spherical shapes of micelles were observed. The size of the micelles was measured at 33 nm, which is smaller than that of the size of micelles from the linear polymer of PS-PI (40 nm) (Figure 3b). At a concentration of 1 mg/mL, spherical and longer objects, such as cylinders or wormlike structures of micelles, are formed (Figure 3c). The diameter of the wormlike micelles was recalculated as 33 nm; these self-assembled into individual discoidal or sunflower micelles. Figure 3d shows micelles only with a wormlike structure that is prepared at a high concentration of PS-PI as 5 mg/mL. The cylinder structure is longer than that obtained using 1 mg/mL, but the diameter of the cylinder is the same (33 nm).

Using the high-pressure emulsification solvent evaporation method, spherical micelles were prepared using poly lactic-co-glycolic acid (PLGA) with 2% polyvinyl alcohol (PVA), 20% sucrose in water, and dichloromethane as the oil phase [61]. The size of the spherical micelles was measured as 158 nm using TEM. Domenico et al. [62] used the SAXS technique to investigate micelles that were prepared using polydimethylsiloxane-b-polyethyleneoxide (PDMS-PEO). The core of the micelle was comprised of a PDMS unit and a shell with a PEO unit. Below the CMC (0.007 g cm^−3^), an initially small micellar unit with a radius of about 2.7 nm was observed. Above the CMC (0.01 g cm^−3^), micellar aggregates with a radius of about 9.5 nm were reported. Further, the size of micelles was found to increase with increasing temperature. Ethylene oxide and propylene-based polymers aggregate and rearrange themselves as micelles with hydrophobics as rigid cores (propylene units) and hydrophilic blocks as coronas (ethylene oxide units) extended into solution. These micelles were investigated using TEM, and the size of the micelles was measured as being between 8 and 15 nm [63]. Micelles using poly(styrene-b-vinylpyridine) (PS-b-PVP) and their behavior in different alcoholic solvents were studied using ellipsometry and DLS measurements. In less-polar solvents, such as propanol, butanol, and pentanol, micelles were observed with collapsed cores of PS and swollen coronas of PVP. On heating, monodispersed spherical-shaped micelles formed, whereas in polar solvents, such as methanol and ethanol, the aggregation of micelles was observed because of the limited solvation of PS blocks in polar solvents [64].

**Figure 4:**
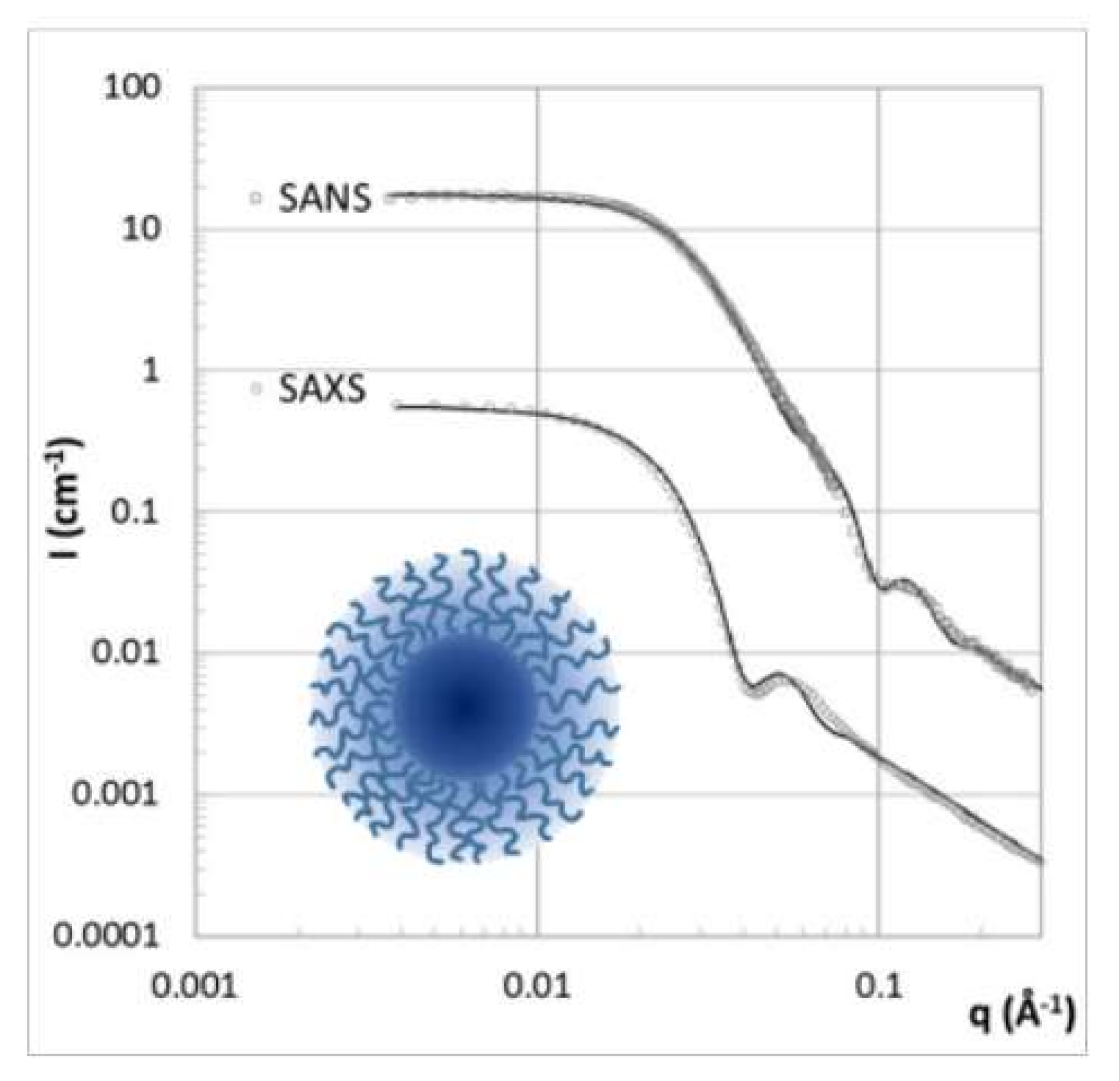
The fit of the SAXS intensity profile of Pluronic (F127) (1%) and linalool (0.2%) solutions in D_2_O at 37 °C. Reprinted with permission from [65]. Copyright 2018 American Chemical Society.

**Figure 5:**
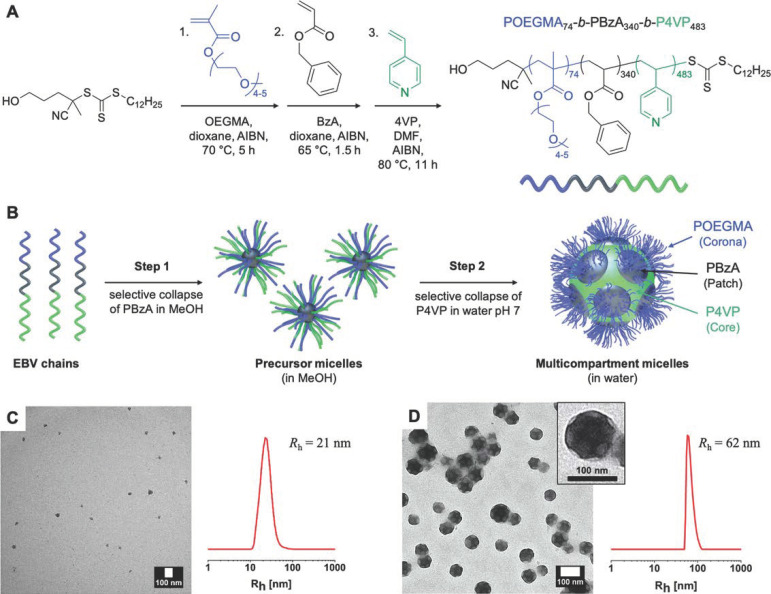
Synthesis of POEGMA-b-PBzA-b-PVP and self-assembly characterization. (**A**) reaction scheme involved in the preparation of the polymer and (**B**) step-wise formation of micelles and multi-compartment micelles. TEM and DLS measurements of polymers in (**C**) methanol and (**D**) in water at pH 7. Reprinted from ref. [66].

SAXS intensities help to understand the properties and to calculate the size of micelles. The SAXS intensity fit of PDMS-PEO at 0.01 g cm^−3^ (above the CMC) was used to calculate the radius of the core and shell. The radius and core were measured as 2.4 and 4.7 nm, respectively. Low statistics of the scattering signal were observed below a CMC of about 0.002 g cm^−3^. This occurred because of the large number of hydrated PEO chains at a lower concentration of PDMS-PEO. SAXS measurements are used for the characterization of micelles prepared using Pluronics F127 and linalool. Figure 4 shows the SAXS intensity profile of F127 and linalool solutions in D_2_O at 37 °C. The shift of the oscillations with a low q was observed because of the swelling; thus, the first oscillation was enhanced, and a second smaller oscillation occurred [65]. As a result, the radius of the core and shell were measured as being 1.2 and 2.6 nm, respectively [62]. Spherical multi-compartment micelles were prepared using a triblock copolymer of Poly(oligo(ethylene glycol) methacrylate-b-poly(benzyl acrylate)-b-PVP) (POEGMA-b-PBzA-b-PVP) that was synthesized by RAFT polymerization [66]. Step-wise self-assembly results in multi-compartment micelles with POEGMA as the coronas, PBzA as patches, and PVP as the cores. Micelles with a size of 21 nm are formed in methanol with the selective collapse of PBzA. These micelles result in multi-compartment micelles by the selective collapse of PVP in water at pH 7 (Figure 5). The formations of precursor micelles and multi-compartment micelles were investigated using TEM measurement (Figure 5). The size distribution of multi-compartment micelles from the TEM images was measured at about 62 nm. This was almost 3 times the size of normal micelles (21 nm); this bigger size is due to the aggregation of normal micelles. This study confirmed that solvent selection and the pH change of the polymer solution will lead to different, self-assembled structures of micelles.

Micelles were prepared using di- and triblock homo- and hetero-chiral block copolymers. The polymer units were comprised of PEG and poly(amino acid) (PAA) blocks with central blocks as poly(aspartic acid) and poly(glutamic acid-γ-hydroxamate). The size of the micelles was measured between 19 and 200 nm by TEM measurement [67]. Using pyrene, the CMC was calculated between 2 and 42 µg/mL for different di- and triblock copolymers. Amino acid compositions in polymer play a vital role in the dimensions of micelles [67]. A triblock copolymer of PEG-b-poly(aspartic acid)-b-Poly(D-leucine-co-tyrosine) was used for the preparation of micelles [68]. Oil-in-water emulsion techniques were adopted for the preparation of spherical micelles with sizes between 30–80 nm. PEG and Poly(D-leucine-co-tyrosine) were used as hydrophilic and hydrophobic units, respectively. The aspartic acid unit in the polymer serves as a cross-linking unit and further helps in stabilizing the micelles.

### 2.2. Polymeric Inverse Micelle

**Scheme 2:**
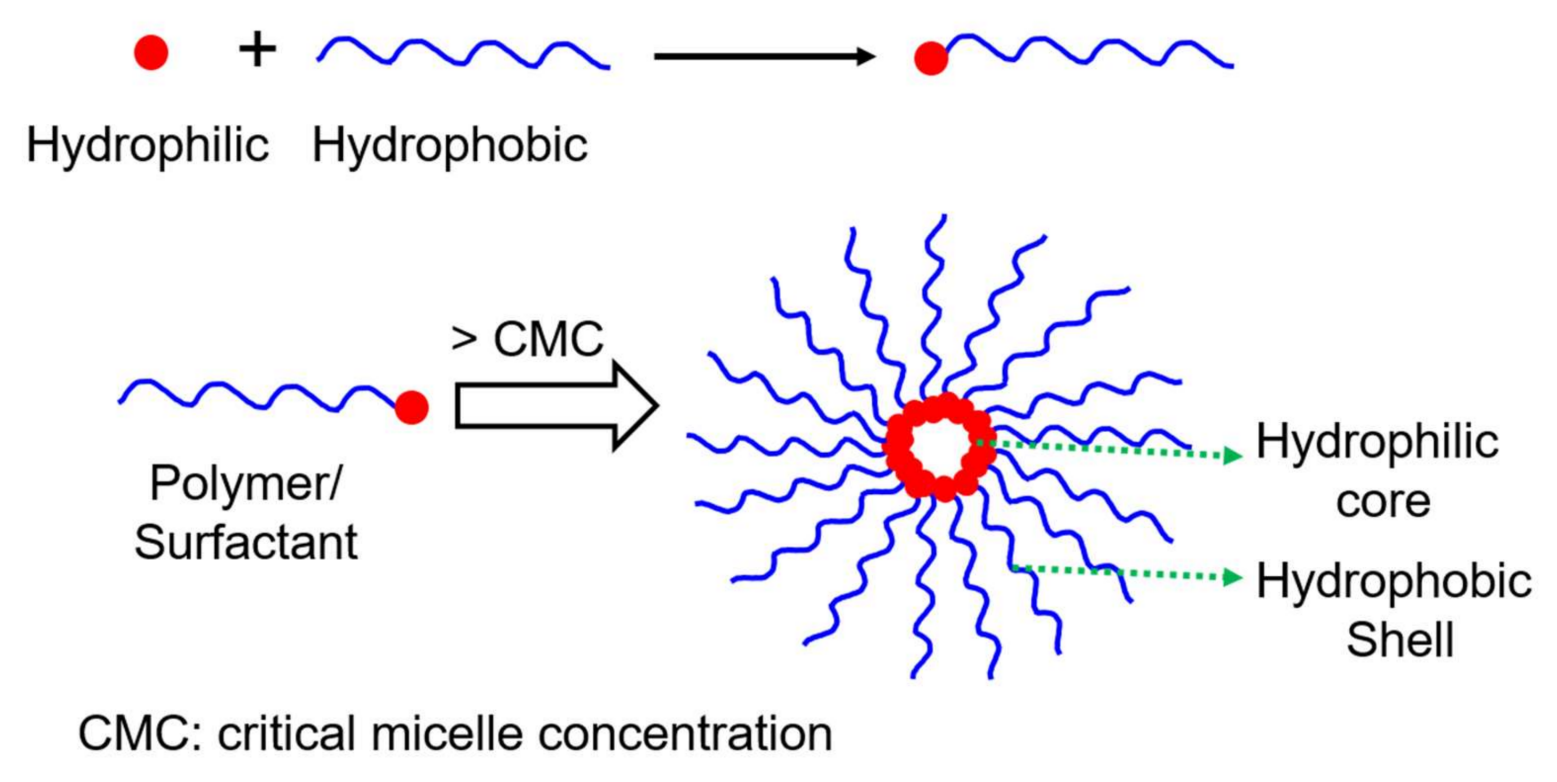
Illustration of self-assembly of a polymer or surfactant into an inverse micelle.

When the hydrophilic head is faced inside while the hydrophobic tails are projected outside, this arrangement refers to a reverse micelle, as shown in Scheme 2 [40,69,70,71]. The inverse micelle can form a nanostructure or undergo self-assembly in non-aqueous solutions [72]. The hydrophilic and hydrophobic parts will interact strongly with polar and non-polar solvents, respectively. Inverse micelles are formed from branched macromolecules [72], water-soluble dendritics [73,74], star-shaped polymers [75,76], and hyperbranched polymers [77,78].

**Figure 6:**
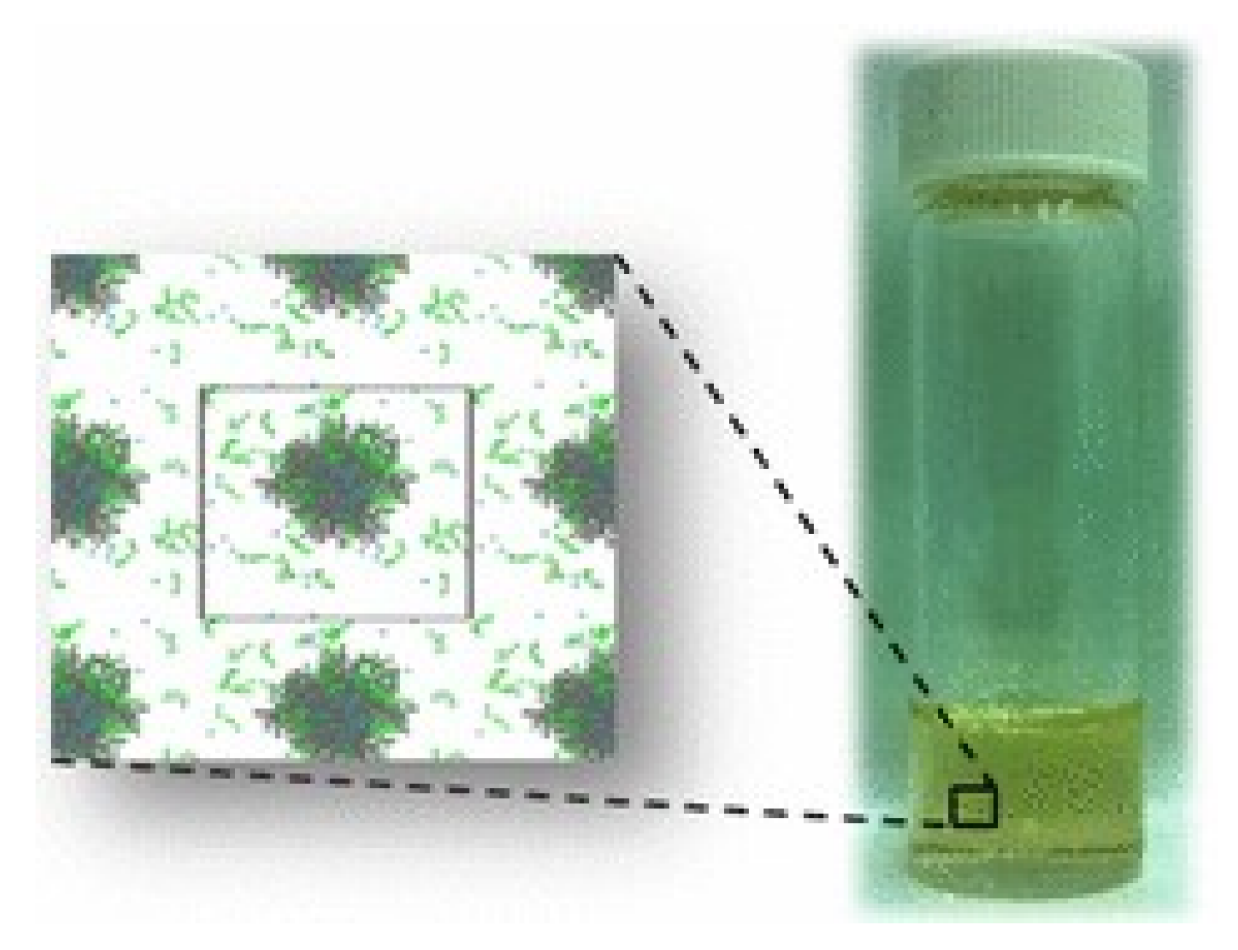
Inverse micelles were prepared in a (**left**) simulation study and (**right**) using the water/oil microemulsion method. Reprinted with permission from [81]. Copyright 2017 American Chemical Society.

Inverse micelles are prepared using cationic, anionic, mixed, zwitterionic, amphiphilic, and nonionic surfactants [78]. Anionic surfactants, such as sulfosuccinic acid bis(2-ethylhexyl) ester sodium salt, are widely used as surfactants in the preparation of inverse micelles [79]. Cetyl-trimethyl-ammonium bromide (CTAB), chloroform, and butyl alcohol were used to prepare reverse micelles [80]. Inverse micelles were prepared using lecithin, monoglycerides, and medium-chain triglycerides in the aqueous phase (Figure 6). The prepared inverse micelles were studied experimentally using DLS and EPR techniques. Further, the results were compared with a molecular dynamic study. The simulation studies revealed the formation of thermodynamic stable micelles from the conformations [81].

In collaboration with undergraduate students, Harris et al. have investigated the use of inverse micelles as templates for the size-controlled formation of nanomaterials through simple, controlled precipitation reactions. A CdSe model was used; further, this was proposed for the investigation of ligand–metal chelation, surface chemistry, growth kinetic, hydrodynamic, and thermodynamic studies [82]. PLA-b-PEG block copolymers were used for the formation of inverse micelles in toluene/ethanol/water as a solvent/co-solvent/water system. The spherical size of the prepared inverse micelle was calculated between 18 and 66 nm [83] by TEM measurement. Star-shaped and linear poly(glycidyl methacrylate) micelles were self-assembled as reverse micelles in organic solvents. The size of the micelles was measured as 20–60 nm using DLS, and the sizes of the micelles changed with the solvents [84]. The micelles’ size did not change with the changing of the solvent from tetrahydrofuran (THF) to dichloromethane (DCM) because both solvents are moderately polar. However, in ethyl oleate, the sizes of the micelles decreased by half of the size of those obtained using THF because of the decrease in solvent polarity [84].

### 2.3. Different Types of Polymeric Micelles

Mixed micelles can be formed from the micellar aggregation of the mixtures of amphiphilic surfactants or polymers [85,86,87]. These mixed micelles exhibit shapes and characteristics different from those produced by individual amphiphilic surfactants and polymers [88]. Mixed micelles find application as drug-delivery nanocarriers, imaging contrast agents, and chemotherapeutic agents [54,88,89,90,91,92,93].

**Figure 7:**
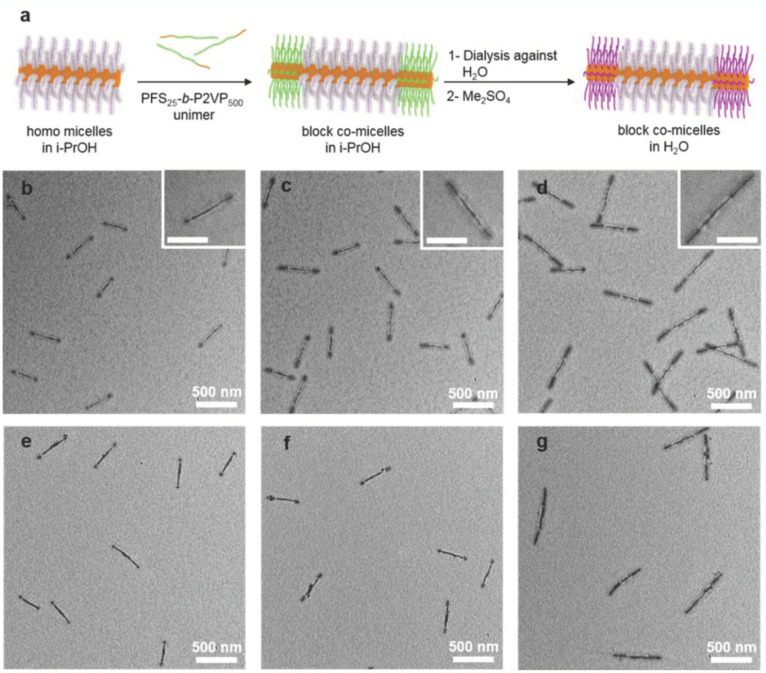
(**a**) Schematic illustration of the preparation of homo-micelles and block co-micelles in isopropanol and water using (PFS_25_-b-P2VP_500_)-b-(PFS-b-(PEO-g-TEG))-b-(PFS_25_-b-P2VP_500_). TEM images of a monodispersed cylindrical micelle of addition of (**b**) 10 µg, (**c**) 20 µg, (**d**) 40 µg of PFS_25_-b-P2VP_500_ unimer to 20 µg of PFS-b-(PEO-g-TEG) cylinders in isopropanol. (**e**–**g**) TEM images of triblock co-micelles (shown in (**b**–**d**) after adding Me_2_SO_4_ and dialysis against water. Reprinted with permission from [95]. Copyright 2016 American Chemical Society.

Triblock copolymer ABA, composed of an A block, such as poly(2-methyl-2-oxazoline), and a B block of either barely hydrophobic poly(2-n-butyl-2-oxazoline) or highly hydrophobic poly(2-n-nonyl-2-oxazoline), self-assembled into a wormlike structure [94]. Cylindrical micelles are prepared using a linear–brush block copolymer of poly(ferrocenyldimethylsilane)-b-poly(allyl glycidyl ether) (PFS-b-PAGE) as a linear chain and a brush of triethylene glycol (TEG), abbreviated as PFS-b-(PEO-g-TEG), a triblock copolymer. Hierarchical self-assembly was observed using a B-A-B-type polymer, where A is the hydrophilic segment PFS-b-(PEO-g-TEG) and the hydrophobic B segment is PFS-b-poly(VP) [95]. Figure 7 shows a broad range of monodispersed cylindrical micelles and co-micelles [95].

Amphiphilic conjugate with a cyclic peptide in between hydrophilic (PEG) and hydrophobic poly(n-butyl acrylate) (PBA) was prepared by RAFT polymerization. The cyclic peptide unit in the polymer forms a large, tubular aggregation into cylindrical micelles in water [96]. The length of the cylinders was between 200 and 400 nm, and the radius of the core measured between 8 and 10 nm. The radius of the core and length of the cylinder-shaped micelles changed with the length of the PBA and PEG units, respectively. pH-responsive polymer, poly(dimethylamino ethyl methacrylate) with cyclic peptide self-assembled into cylindrical micelles by hydrogen bonding. These cylindrical micelles can be reversed to dispersed polymer units with a change in pH [97]. pH controls the assembly of poly(hydroxyethyl acrylate) and PAA with cyclic peptides. The cylindrical micelle was characterized using the DLS, TEM, and SANS techniques. DLS revealed the diameter of the micelles as measuring between 15 and 800 nm with a change in solvents, DMF, TFA, methanol, and water. The cylinders’ lengths and diameters were measured by TEM as 50–100 nm and 8–12 nm, respectively. The increase in hydrophilic units results in a hydrodynamic diameter measured by DLS as 150–250 nm; TEM showed 100–250 nm in length and 8–12 nm in width. In DMF, SANS revealed them to be 23 nm in length and 10 nm in diameter, but in water, the length was elongated to 65 nm [98].

Bottlebrush copolymers were prepared using methoxy oligo(ethylene glycol) methacrylate and alkoxy oligo(ethylene glycol) methacrylate by RAFT polymerization in toluene. This forms flower-like micelles with hydrophobic cores and hydrophilic shells in water. A hydrophobic, alkoxy oligo(ethylene glycol) unit serves as the backbone, and the hydrophilic, methoxy oligo(ethylene glycol)-based units form the linear chains [99].

## 3. Application of Polymeric Micelles

Polymeric micelles are novel drug vehicles that present numerous advantages, such as the reduced side effects of drugs, selective targeting, stable storage, and stability toward dilution [80,81,83,95,97]. Furthermore, polymeric micelles possess a nanoscale size with a narrow distribution [100,101]. Micelles can shield drugs against oxidation in vitro and in vivo, owing to their core–shell structure [80,83,95]. More importantly, polymeric micelles can be fabricated with appropriate drug molecules [80,81,83,95,97]. In this section, the applications of micelles are discussed.

### 3.1. Cancer Drug Delivery

Micelles prepared using PVP-b-PVAc showed a clofazimine drug-loading capacity of about 20 wt% in vitro experiments against breast cancer cells MCF12A and MDA-MB-231. The size of the micelles after the loading of clofazimine in PVP90-b-PVAc290 was about 210–220 nm and was stable for 16 h in PBS buffer solution at pH 7, 34 °C [59]. Wormlike micelles of triblock copolymers with poly(2-methyl-2-oxazoline) and poly(2-n-butyl-2-oxazoline) or with highly hydrophobic poly(2-n-nonyl-2-oxazoline) deform into a raspberry micellar structure. This deformation of structure takes place on the loading of a cytostatic drug (PTX) [94]. Curcumin-loaded PLGA micelles showed entrapment efficiency of about 46.6%. This drug-loaded PLGA was stable for 1–2 months at 4 °C, and the oral bioavailability of curcumin in PLGA was measured as being 22-fold higher than conventional curcumin [61]. Daunorubicin was loaded using PEG-PAA-based triblock copolymer micelles [67,68]. Using homo-chiral and hetero-chiral PEG-PAA-based copolymers, a maximum efficiency of about 97.7% was obtained for the loading of Irinotecan [67]. Using PEG-b-poly(aspartic acid)-b-Poly(D-leucine-co-tyrosine), hydrophobic drugs were loaded. pH-dependent drug releases were studied systematically. Anti-cancer drugs were loaded and released into solid tumors [68]. Triblock copolymers with PEG (hydrophilic block), a glutamic acid hydroxymate unit as a central block that interacts with iron atoms by a coordination bond, and a hydrophobic block of polypeptide was used as a probe for loading anti-cancer drugs, such as SN-38, danorubicin, epothilone D, aminopterin, paclitaxel, and panobinostat. Loaded active pharmaceutical ingredients were about 80% efficient, and the average size of the micelles was calculated as being between 58 and 120 nm using TEM and DLS. Magnetic resonance imaging (MRI) relaxivity studies revealed spin–lattice and spin–spin relaxivity as 7–16 and 36–53 mM^−1^ s^−1^, respectively [101].

**Figure 8:**
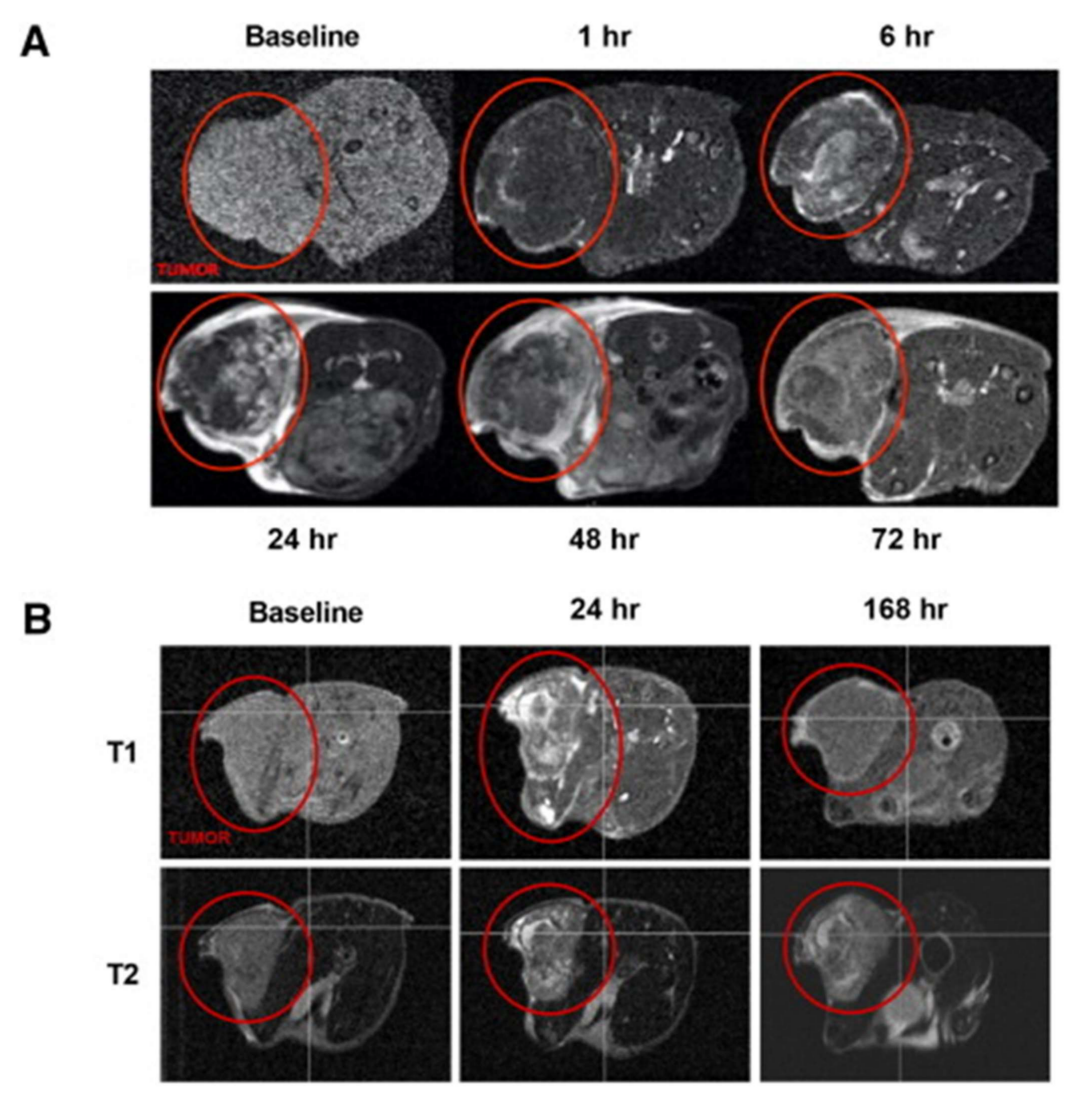
MRI imaging of iron-stabilized micelles in mouse (**A**) MRI of HCT16 mouse xenograft (1–72 h) and (**B**) (24–168 h). The red circles indicate tumors. Reprinted from [101], Copyright (2017) with permission from Elsevier.

Figure 8 reveals the MRI study results using iron-stabilized micelles in the tumor model of HCT116. An HCT116 mouse xenograft model post-administration with drug SN-38 shows a contrast image (Figure 8A). Iron-stabilized micelles in tumors peaked between 24 and 48 h, and the signal was cleared at 168 h, as obvious from Figure 8B. This is evidence for the use of an iron-stabilized triblock copolymer as a promising MRI contrast agent [101]. The 90% loading efficiency of epothilone D drug was obtained using triblock copolymers (IT-147). The drug-loaded micelles were about 75 nm in diameter and showed pH-dependent drug release without enzymatic activation. IT-147 showed a level of epothilone D in the plasma compartment that was 6-fold greater than that of the free drug [100].

### 3.2. Another Application

Inverse micelles are widely used in food technologies, such as the extraction of protein and enzymes [102]. Reverse micelles were prepared using bis-(2-ethylhexyl)sulfosuccinate sodium to separate α-glucosidase from mouse intestine extract by countercurrent chromatography. This micelle is proposed for protein separation [79]. CTAB was consumed for the preparation of reverse micelles and used for protein extraction from grape seeds. The yield of protein extraction from the grape seed was calculated as 82.3% [80]. A cylindrical micelle was used for the complexation of DNA through a “click” reaction [95]. Diblock copolymers, with PEG used as one block, and the other block composed of either poly(D, L-lactide) (PDLLA), copolymers of poly(D, L-lactide-co-caprolactone) (PDLLACL), or poly(glycolide-co-caprolactone) (PGACL), were prepared using bulk ring-opening polymerization. PDLLACL–PEG resulted in an optimum polymer for the solubilization of paclitaxel compared to the PDLLA–PEG polymer. Micelles prepared using PDLLA–PEG showed a 95% dissociation of loaded paclitaxel in rat blood within 15 h [103]. An amphiphilic brush copolymer, composed of poly(L-lysine)-g-(oligo(γ-benzoyl-L-glutamate)-b-PEG (PLL-g-(PBLG-b-PEG)) or PLL-g-(oligo(ε-benzyloxycarbonyl-L-lysine)-b-PEG) (PLL-g-(PZLL-b-PEG), were synthesized by ring-opening polymerization. PLL served as a backbone, while PBLG-b-PEG or PZLL-b-PEG served as a side chain. The unimolecular micelles with this amphiphilic brush polymer were prepared with a hydrophobic probe used for loading pyrene and oil red [104].

**Figure 9:**
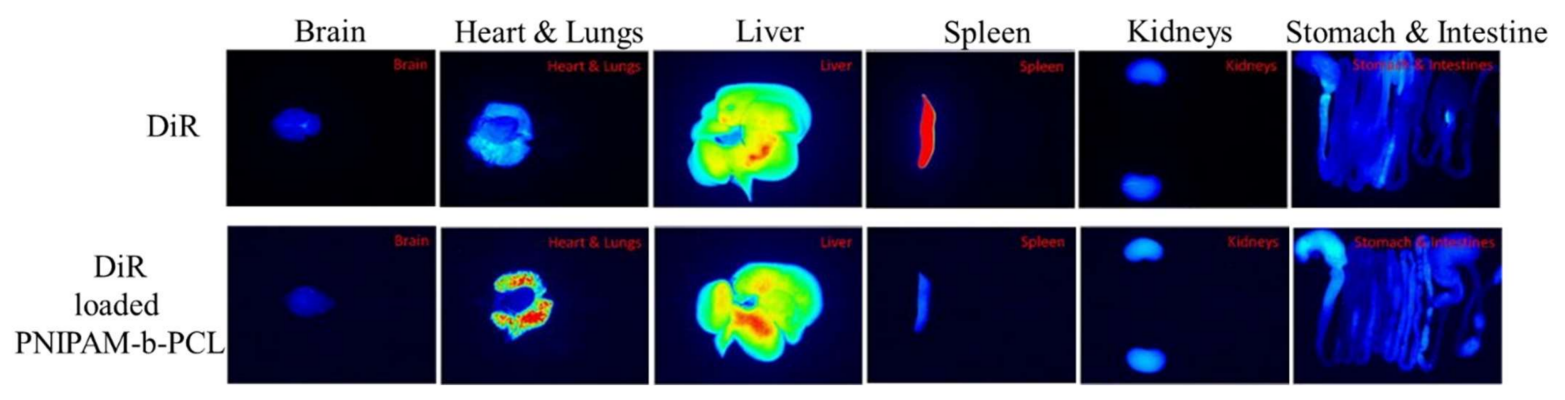
Fluorescence imaging of rat organs using DiR solution and DiR-loaded PNIPAM-b-PCL, reprinted from ref. [106].

Thermoresponsive copolymer micelles were prepared using poly(N-isopropylacrylamide)-b-poly(ε-caprolactone) (PNIPAM-b-PCL) amphiphilic polymer [105]. This thermoresponsive micelle is used for bioimaging applications. Dioctadecyl-3,3,3′,3′-tetramethylinotricarbocyanine iodide (DiR) was used as a control for imaging rat organs. DiR-loaded micelles of PNIPAM-b-PCL showed improved signals in the lungs, kidney, stomach, and intestine compared with DiR solution (Figure 9) [106].

**Figure 10:**
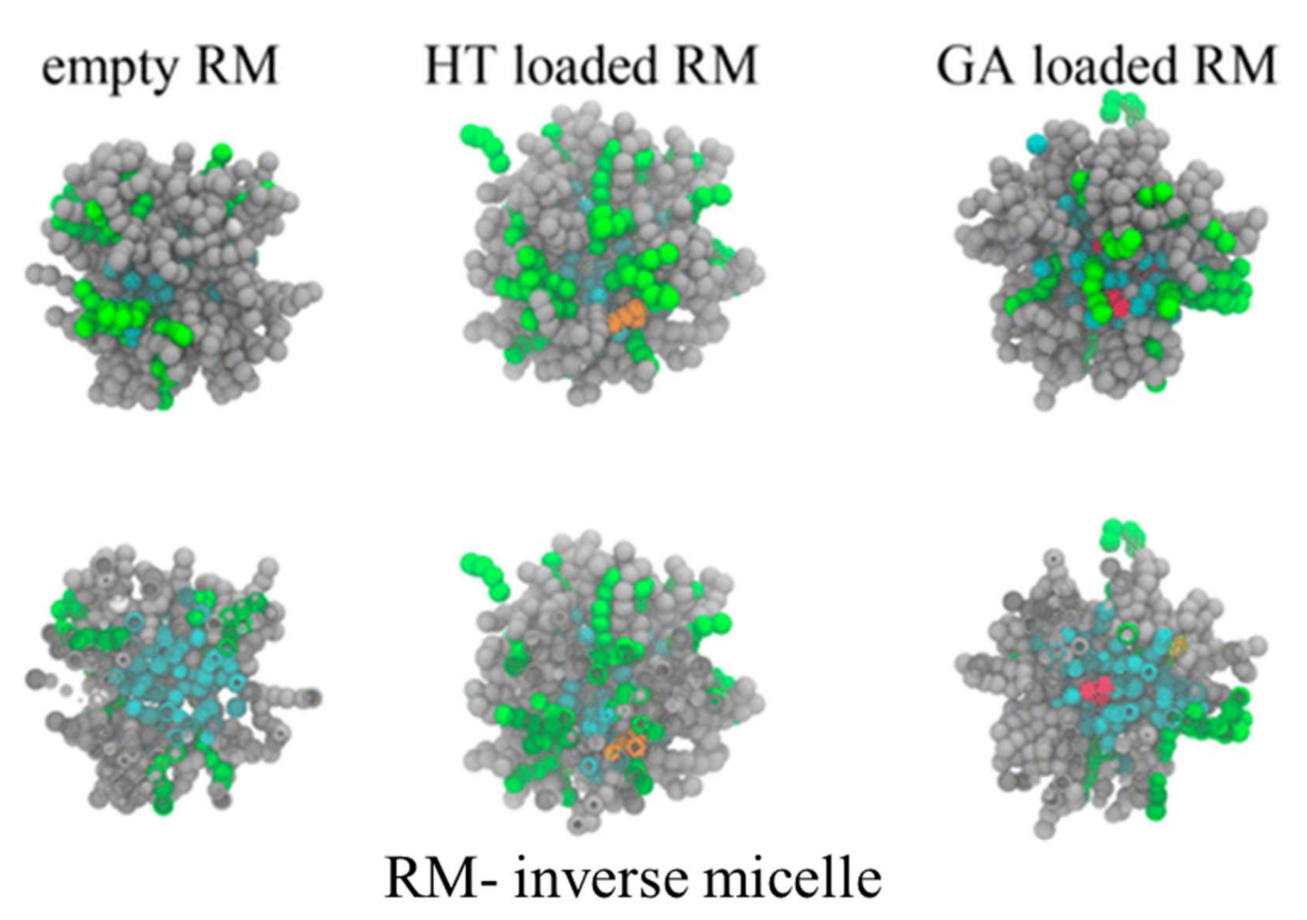
IT-147 clusters of inverse micelles produced by the simulation study: empty RM (without antioxidant), HT-loaded RM, and GA-loaded RM are antioxidant-loaded RMs. Reprinted with permission from [81]. Copyright 2016 American Chemical Society.

Inverse micelles prepared using the water/oil microemulsion method were compared with inverse micelles formed via simulation studies [81]. The prepared inverse micelles were used as antioxidant (hydroxytyrosol (HT) and gallic acid (GA)) nanocarrier probes. Experimentally, using DLS, the size of the micelles was calculated as being 20 nm without HT or GA. The size of the micelles with GA and HT were measured as 30 and 20 nm, respectively. By EPR, the mobility of the spin probe increased slightly (2.20 ns) with HT than without HT (2.19 ns). At the same concentration, the mobility of the spin increased to 2.23 ns in the presence of GA. This increase was caused by the interactions of the GA with ethanol, and because of this swelling, the greater size of the micelles with GA was observed using DLS measurements. Further, the presence of HT at the core and GA in the shell were confirmed via EPR and simulation studies. Figure 10 shows the clusters of inverse micelles isolated by molecular dynamic studies. An HT (orange color) molecule lies at the core of the micelles; GA (red color) lies at the shell of the micelles. (Water molecules are represented in blue).

**Figure 11:**
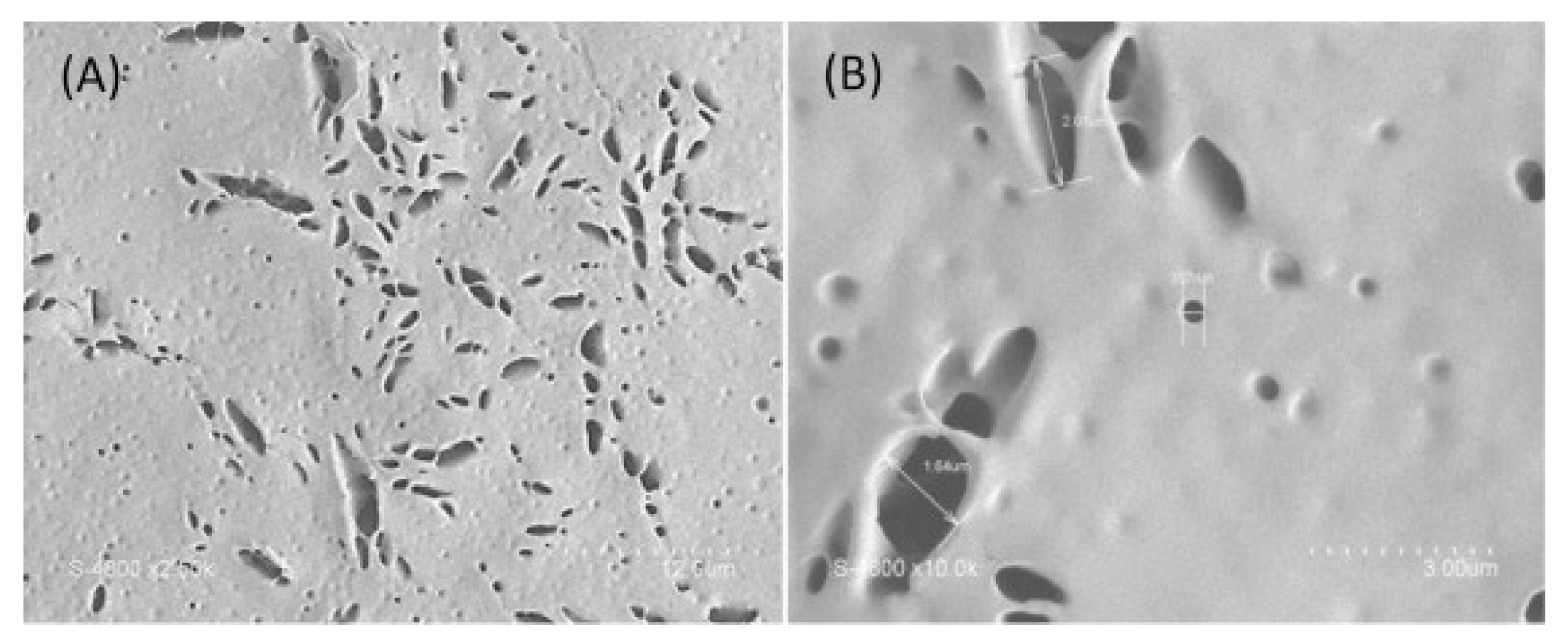
SEM images of PLA-b-PEG-b-PLA inverse micelle membrane with heparin in different magnifications (**A**) higher magnification and (**B**) lower magnification. Reprinted from [83], Copyright (2015) with permission from Elsevier.

PLA-b-PEG-PLA inverse micelles were used for loading the hydrophilic drug heparin [83]. A total of 50% of the heparin was released from the micelles within 4 h, and within 24 h, the almost-complete release of heparin was observed. The swelling of the polymer in PSB opens the membrane pores, as shown in SEM images (Figure 11). The porous structure of the membranes has a diameter ranging from 100 to 2000 µm; however, this is much greater than that of inverse micelles (~60 nm). This is caused by the aggregation of the inverse micelles in the membrane. Upon immersion in PBS, the membranes were hydrated, leading to the opening of the membrane, thus releasing heparin efficiently.

AB and AB_3_-types of block copolymers with hydrophilic A blocks of poly(sarcosine) and hydrophobic B blocks of poly(L-lactic acid) were prepared using the ring-opening polymerization method. The N-terminals were arranged using guanidium or guanidine-peptide nucleic acid for the loading of the siRNA. By DLS, the diameter of the micelles was measured as 25 nm after the loading of the siRNA. The water molecules in the core only numbered about 5 × 10^5^ molecules, suggesting that they were insufficient to stabilize the ionic molecules inside inverse micelles. Guanidium or guanidine-peptide nucleic acid are involved in the ion-complex with siRNA, and thus the size of the micelles was reduced after loading [107]. Poly(hydroxyethylaspartamide) (PHEA) with PEG and hexadecyl amine (C16) forms micelles. Among micelles of PHEA-C16 and PHEA-PEG-C16, the PHEA-PEG-C16 micelles showed promising results as nanocarriers for the ocular delivery of the drugs netilmicin sulphate, dexamethasone alcohol, and dexamethasone phosphate [105]. In an ex-vivo study, poloxamer micelles loaded with iodine reduced the permeation of iodine through the skin, as compared to povidone-iodine [108]. Associative nano-emulsions were prepared using triblock amphiphilic polymers (PEO-PCL-PEO) with lecithin. This was characterized by dipole–dipole interactions between the PEO blocks and the phosphorylcholine group (hydrophilic head) of lecithin. By rheological and water evaporation measurements, the water evaporation behavior of the associative nano-emulsion was determined. A short PEO chain enhanced the skin penetration of the nano-emulsion, as compared to a longer PEO chain [109]. Berberine-loaded thiolated F-127 polymeric micelles (BTFM) and berberine-loaded F-127 micelles (BFM) were prepared using the thin-film hydration method. According to skin permeation and retention studies, BTFM showed a decreased transdermal amount and increased skin retention as compared to BFM. This is because BTFM shows a strong affinity with keratin, which sustained the drug release [110].

## 4. Summary and Outlook

Recently, polymeric micelle molecules have been broadly studied for different applications. In this review, articles related to micelles, types of micelles, and applications of micelles, were extensively reviewed. The formation of CMCs and the investigation of CMCs using the surface-tension method, capillary method, Wilhelmy plate method, filter method, and dye micellization method were discussed in detail. Methods of preparing micelles are discussed briefly, with a lengthy discussion of the types of micelles. Spherical micelles, inverse micelles, mixed micelles, cylindrical, and bottlebrush-shaped micelles were discussed. Micelles are used in bio-applications because of their unique properties, including biocompatibility and their capability to protect drugs, target drug delivery, increase the circulation of a drug, and so on. In the last section, the applications of micelles were discussed, which mainly include drug delivery applications, and imaging, ocular delivery, and skin treatments.

In the future, research on micelles needs to focus on the preparation of different shapes of micelles. Different shapes might help target drug-delivery applications. The disadvantages of micelles include the poor solubility of small-sized micelles, poor loading capacity, and poor physical stability in vivo; these need to be overcome. The application of micelles is limited to drug delivery; in particular, extensive research on imaging and skin treatments using micelles is necessary. The mechanism of micelles in drug-delivery applications is in the development stage, thus requiring comprehensive analysis. These improvements will help in the development of use for micelles in clinical applications.

## Data Availability

No supporting data.
